# Shape classification technology of pollinated tomato flowers for robotic implementation

**DOI:** 10.1038/s41598-023-27971-z

**Published:** 2023-02-07

**Authors:** Takefumi Hiraguri, Tomotaka Kimura, Keita Endo, Takeshi Ohya, Takuma Takanashi, Hiroyuki Shimizu

**Affiliations:** 1 Faculty of Fundamental Engineering, Nippon Institute of Technology, Saitama, 345-8501 Japan; 2 Faculty of Science and Engineering, Doshisha University, Kyoto, 610-0321 Japan; 3 Kanagawa Prefectural Agricultural Research Center, Kanagawa, 259-1204 Japan; 4 Forestry and Forest Products Research Institute, Tohoku Research Center, Iwate, 020-0123 Japan

**Keywords:** Plant development, Plant reproduction

## Abstract

Three pollination methods are commonly used in the greenhouse cultivation of tomato. These are pollination using insects, artificial pollination (by manually vibrating flowers), and plant growth regulators. Insect pollination is the preferred natural technique. We propose a new pollination method, using flower classification technology with Artificial Intelligence (AI) administered by drones or robots. To pollinate tomato flowers, drones or robots must recognize and classify flowers that are ready to be pollinated. Therefore, we created an AI image classification system using a machine learning convolutional neural network (CNN). A challenge is to successfully classify flowers while the drone or robot is constantly moving. For example, when the plant is shaking due to wind or vibration caused by the drones or robots. The AI classifier was based on an image analysis algorithm for pollination flower shape. The experiment was performed in a tomato greenhouse and aimed for an accuracy rate of at least 70% for sufficient pollination. The most suitable flower shape was confirmed by the fruiting rate. Tomato fruit with the best shape were formed by this method. Although we targeted tomatoes, the AI image classification technology is adaptable for cultivating other species for a smart agricultural future.

## Introduction

Smart agriculture actively incorporates engineering and chemical technologies in agriculture, especially crop cultivation. Smart agriculture aims for labor saving and high-quality production^1,2^. In particular, the digitized data (such as temperature, humidity, sunshine hours, and soil components) assist cultivation management by providing information and communications technology (ICT) and the Internet of Things (IoT)^3–7^. For example, physiological and physiological plant growth models can provide optimal methods^8^. However, the parameters interact with complex control variables. These parameters vary widely, including nutrients, flooding, photosynthetic light intensity, and carbon dioxide. Moreover, changes in plant species and environmental conditions lead to interactions between these parameters, limiting independent utility.

Therefore, to improve smart agriculture, we propose using artificial intelligence (AI) with machine learning^9,10^. Practical AI are already in operation in plant factories such as hydroponics^11^. For example, it is useful when cultivating in artificially controlled environments to optimize the water and air temperature and light intensity. AI is also used to improve soil management and analyze nutrient levels^12–14^. Furthermore, advanced technology is underway to use robots with AI to support cultivation to reduce labor costs. For example, pesticide application^15^ and automated harvesting^16,17^.

Tomato is a high demand and popular cultivation crop. Tomato ranks first in crop production in the world. A total of 1.8 million tons of tomato are produced annually, followed by onions (1.04 million tons) and cucumbers (0.91 million tons)^18^. Tomato are the most popular crops but have many cultivation problems, including pollination^19^. Pollination involves the pollens being created in the stamens, which pollinates the pistils to bear seeds and fruits. Cross pollinated plants must be pollinated by pollen from different strains. However, tomatoes can easily be pollinated within a single flower once pollen is created. Hence, tomatoes are self-pollinated when flowers are shaken. In tomato greenhouse cultivation, pollination schemes commonly use three methods. These are pollination using insects, artificial pollination (by manually vibrating flowers), and hormonal pollination using plant growth regulators.

**Figure 1:**
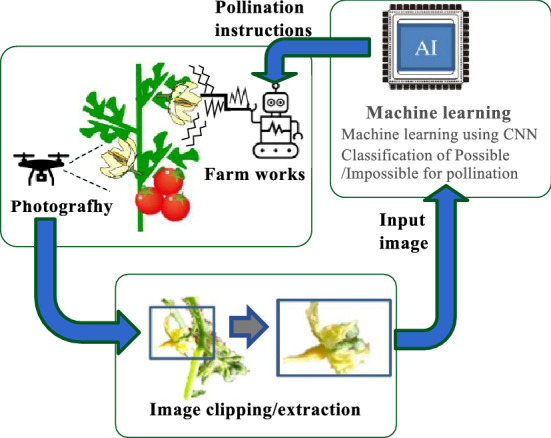
Pollination system using AI.

Pollination using insects follows nature. Tomato flowers are pollinated by shaking of the flowers when honeybees and bumblebees collect pollen. However, insect management and rearing are difficult and bees are inactive in high temperatures, reducing pollination efficiency. Therefore, artificial pollination is often used to manually vibrate flowers. In artificial pollination, farm workers visually classify the shapes of flowers that are ready for pollination and shake them using a vibrating instrument. However, farm workers require experience and skills to classify flowers. Hence, many skilled farm workers are required, increasing expenses. Hormonal pollination involves plant growth agents, forcing fruiting, growth, and pollination. Hormonal pollination is an easy and useful technique that does not required experience or skills in classifying flowers^20^. However, if strict guidelines are not followed then phytotoxicity occurs, resulting in quality problems such as deformation of the fruit and limited taste. We propose new pollination schemes and have developed a system to solve these problems (see Fig. 1). The proposed pollination system uses small drones or robots instead of bees and humans. However, this is a complicated process.

For example, drones and robots must be able to find flowers like bees and humans do. Drones and robots require the ability to discriminate ripe flowers. Communication technology for remotely controlling drones and robots is required. A mechanism for pollinating flowers is also required. We focus on the technologies for drones and robots to distinguish flowers autonomously. Furthermore, the robots need to distinguish flowers and also identify the detailed shape of flowers that are ready for pollination.

Drones and robots equipped with cameras captured image data and classified the flowers. We developed an AI image classification system using a convolutional neural network (CNN) based on machine learning. Drones and robots classify the flowers while moving. For example, flowers are shaking due to the wind caused by the drone or the vibration caused by the robot. Movement impacts the analytical performance of the AI image classifier. Therefore, it is necessary classify the shape of flowers in conditions and environments involving drones and robots. We aim to develop the technology to be able use drones and robots to support cultivation by using complicated state-of-the-art technology. It is most important that the technology is effective and meets the requirements of both plant characteristics and actual cultivation conditions. Therefore, we have developed the technology and confirm the usefulness of this AI image classification system in a greenhouse experiment, resulting in fruiting tomatoes.

## Methodology

This section mainly describes basic AI development techniques for robots to classify flowers. To classify pollinable flowers, we used a machine learning CNN algorithm^21^, which is generally used for image analysis. We created an AI image identification device as the basic technology for a pollination system that does not require human involvement, such as visual inspections or managing insects. The CNN techniques used in this study are detailed below. Using the flower classification criteria and CNN algorithms described in this section, the next section leads to mounted technology suitable for pollination robots.

### Flower shape machine learning

**Figure 2:**
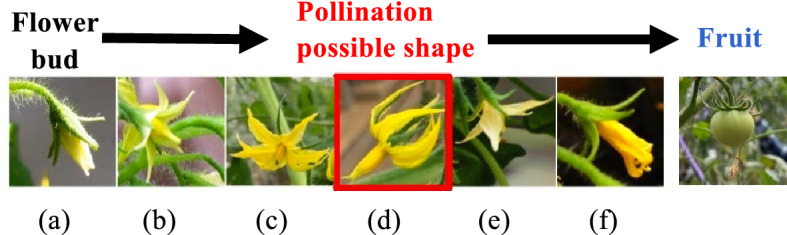
Flowering process and conditions for determining flower shapes of flowers ready for pollination. The process of tomato flower budding, flowering, and fruiting. The process from (**a**) to (**f**), (**a**) is the start of a bud, and fruits grow after (**f**).

The process of tomato flower budding, flowering, and fruiting is provided in Fig. 2. The process from (a) to (f), (a) is a bud, and fruits are grown after (f). The shape of flowers varies during the process of flower bud differentiation. A general empirical rule is that the shape of a pollination flower has petals curled in (d). The stamen protrudes from the center of the flower. Pollen is attached to the inside of the stamens. The stigma of the pistil is surrounded by stamens.

**Figure 3:**
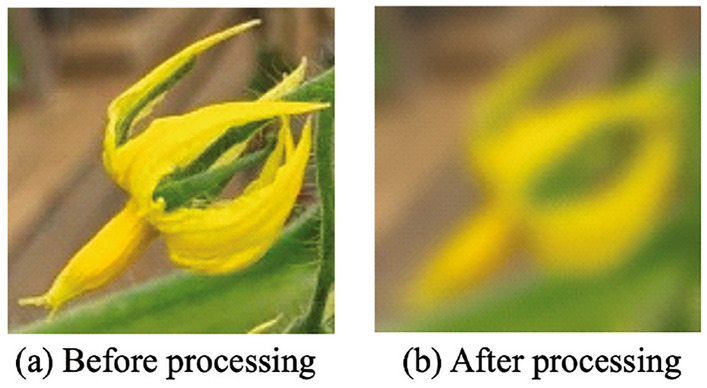
Gaussian filter. (**a**) Is the image before applying Gaussian filter. (**b**) Is the image after applying Gaussian filter.

The shape (d) produces the most pollen when the stigma is long, increasing the probability of pollination. Tomatoes are self-pollinated, when the stamen vibrates, pollen adheres to the stigma and pollination begins. Therefore, the AI image classifier identifies shape (d) using the captured image. Assessing the images taken by drones and robots need to account for the additional shaking by the drone or robot. To simulate image blurring, the image was smoothed using a Gaussian filter. An example image after Gaussian filtering is provided in Fig. 3. We developed an AI image classifier using both normal images and smoothed images for the CNN machine learning.

### Ingenuity of CNN in image analysis

**Figure 4:**
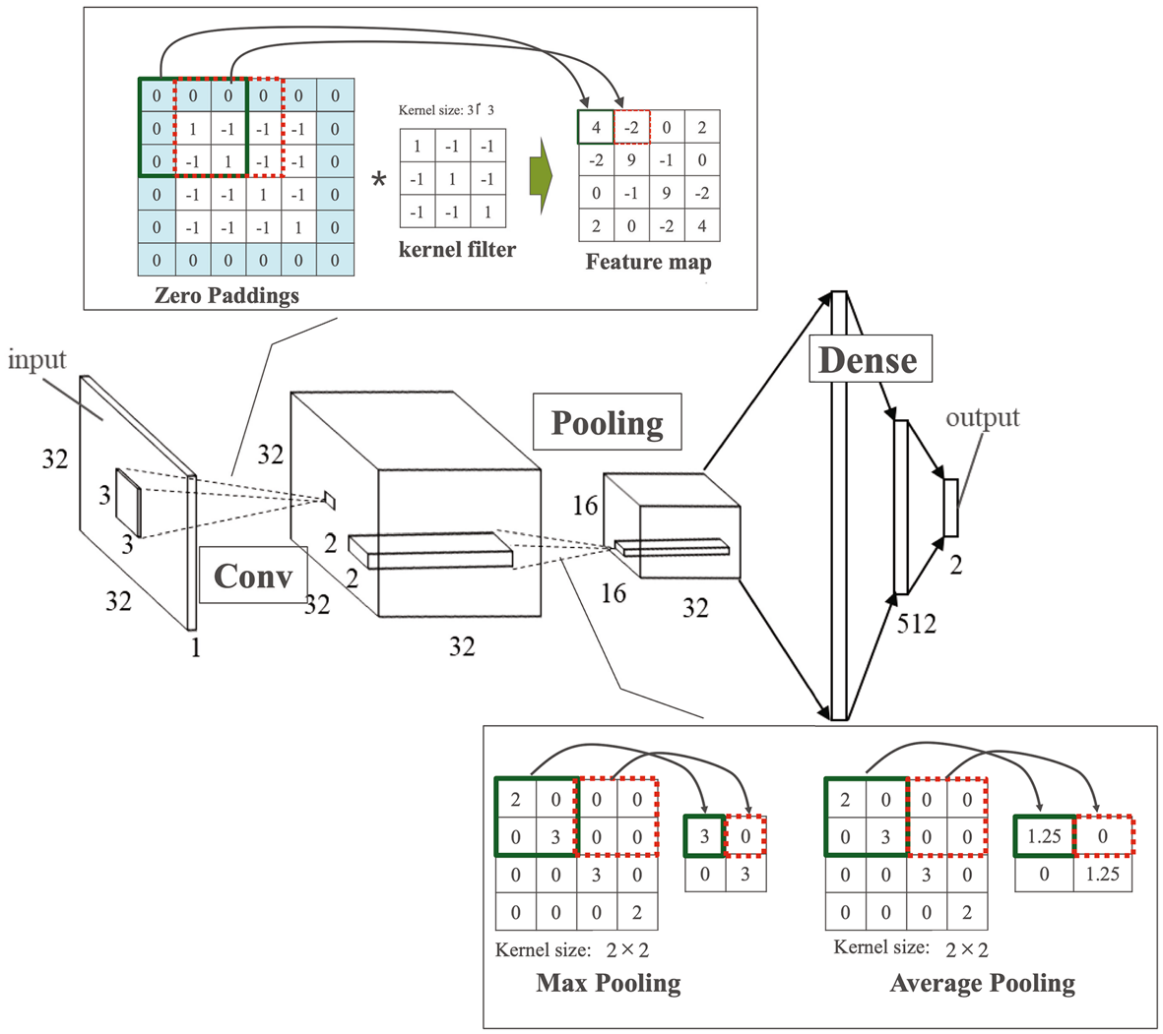
Configuration of CNN. The figure numbers represent the pixel size of the image. Conv performs convolution processing on the image data converted into a cubic matrix of red/green/blue (RGB) and extracts the feature map.

The CNN used in this study was neural networks, extracting features using a convolution (Conv) layer and a Pooling layer. A configuration example using CNN is provided in Fig. 4. The figure numbers represent the pixel size of the image. Conv performs convolution processing on the image data converted into a cubic matrix of red/green/blue (RGB) and extracts the feature map. To extract features (such as edges), zero padding was performed by adding 0 surrounding the image of 32 × 32 pixels. Moreover, a feature map was obtained by shifting the 3 × 3 window one pixel at a time while applying a kernel filter. The maximum value pooling and average value pooling were performed in the pooling layer to counteract image shifts and differences in appearance. Next, Dense (connective layer) weighed the extracted features and transformed them into a one-dimensional vector. Finally, Output (output layer) calculated each classification using an activation function, such as a softmax function. CNN is mainly used in image analysis because of these functions^9^.

**Figure 5:**
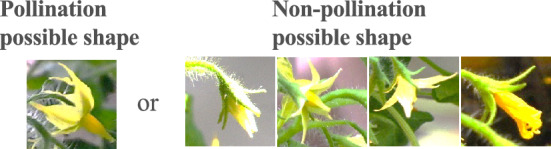
A two class classification system.

CNN algorithms require learning using a large amount of image data. However, it is difficult to collect a large amount of image data, so we padded the data by rotating or reversing the images. The AI image assessment used two types of flower image data to identify flowers ready for pollination and unripe flowers. After learning the two-class classifications, the accuracy rate of the classification results was assessed (see Fig. 5). The training image data involved the original image of 4608 × 2592-pixels. From these images, 100 images were prepared by removing the flower parts and dividing the images into six stages, from bud emergence to the initiation of fruiting.

**Figure 6:**
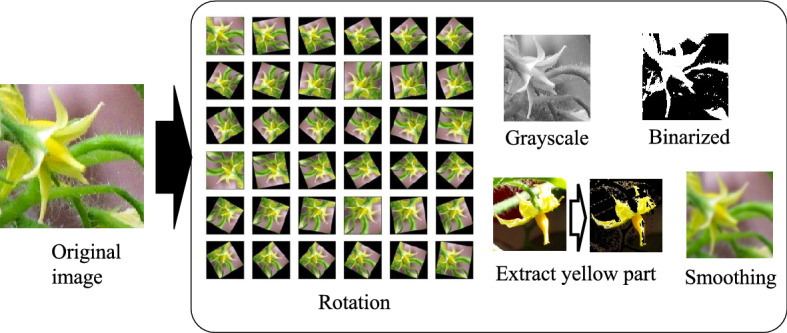
Inflation and pretreatment.

The image size was condensed to 32 × 32 pixels with an average size of 100 KB. In addition, the padding and preprocessing of the training image data was performed, including rotations, grayscale conversions, binarizations, and preprocessing with a Gaussian filter (see Fig. 6). Processing to extract yellow from the image was also performed to learn the flower shape. At the time of learning, the number of training images was padded to approximately 85,000 by dividing them into training images and test images before performing preprocessing. Images of flowers with the high possibility of pollination were extracted to calculate the most accurate rate of pollination by the AI image classifier (see Fig. 2). Moreover, we confirmed the accuracy rate of the smoothed images (representing blurring by movement). Our evaluation assessed the accuracy rate (validation accuracy), which is the percentage of successful discrimination to test additional images not included in the initial image database.

### Ethics declarations

The use of plants in this study complied with relevant institutional, national, and international guidelines and legislation.

**Figure 7:**
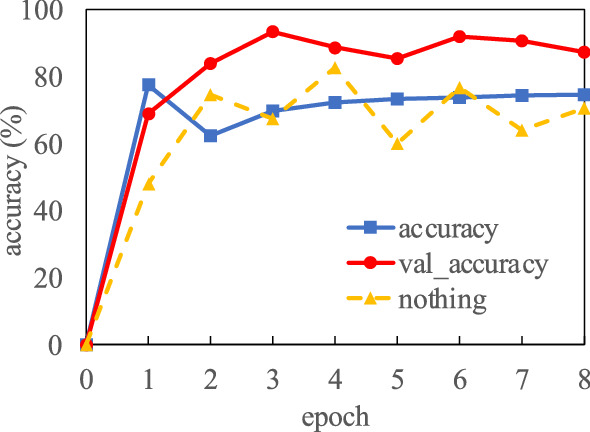
The learning results. The vertical axis provides the accuracy rate, and the horizontal axis is the number of epochs. The red line indicates validation accuracy (val_accuracy), and the blue line indicates training accuracy (accuracy).

**Table 1:** Probability of judging that pollination is possible (%).

	(b)	(c)	(d)	(e)
Normal image	5.0	60.0	96.9	67.4
Image of gaussian filter	4.1	54.7	95.9	63.5

## Results

### Evaluation of CNN machine learning

The analysis of the AI classification algorithm is provided in Fig. 7. The vertical axis provides the accuracy rate, and the horizontal axis is the number of epochs. The red line indicates validation accuracy (val_accuracy), and the blue line indicates training accuracy (accuracy). The experimental results converged at eight epochs (due to early stopping) and the validation accuracy was 87.3%. One of the reasons for the low accuracy rate (of $\le$ 90%) is the insufficient number of original training images.

However, our result is a trade off as we decided it is preferable to determine the operation accuracy rather than focus on an extreme accuracy of the pollination images. Therefore, the accuracy rate may decrease due to images which were difficult to identify between ripe flowers and their boundaries. In contrast to the red line of val_accuracy, the yellow dashed line (nothing) is the validation accuracy when learning without data augmentation as shown in Fig. 7, but the accuracy rate was as low as approximately 70%. Therefore, we confirmed the effectiveness of padding. Table 1 shows the probability of the AI classification algorithm identifying pollination using the shape of the flower cluster. The flower shape of (d) was effectively ripe with approximately 97% accuracy. This technique provided the most accurate results, so the accuracy of image is sufficient. In addition, the smoothed images using the Gaussian filter produced an accuracy rate for the flower shape (d) of approximately 96%. This result suggests the AI remains effective even with shaking caused by drones and robots.

### AI image classifier suitable for robot mounted technology by experimental results

**Figure 8:**
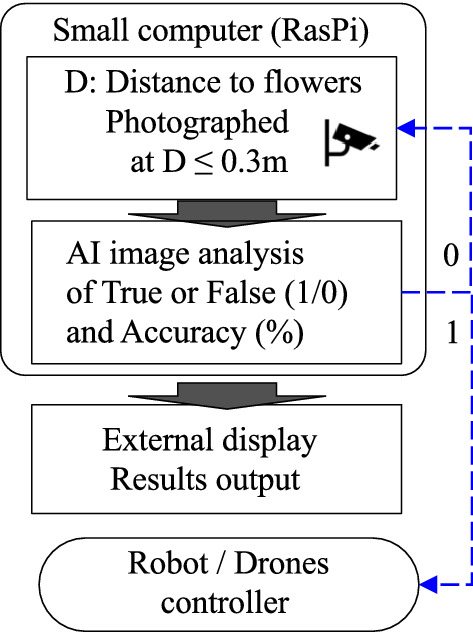
Operation and configuration of the AI image classifier.

We verified the accuracy of the image classification algorithm using real flowers in the tomato growing greenhouse. The image classification algorithm was used in a small computer as an AI image classification machine. The small computer used was a Raspberry Pi (hereafter RasPi)^22^, a single-board computer equipped with an ARM processor. The configuration and operation of the AI image classification machine is provided in Fig. 8. The RasPi was connected to a camera module, an ultrasonic sensor module, and an external LED display that outputs the results. The ultrasonic sensor measured the distance between the flower and RasPi to link the camera to take an image. Hence, the image was only automatically taken when the tomato stem was within 0.3 m of the camera. Since this function eliminates the need to take extra images and obtains only image data with a fixed view angle, the flower parts can be easily extracted. The output of the AI image classification machine displays an accuracy rate (%) on the external LED display. It also states outputs 1 when it determines pollination is possible, and outputs 0 when it determines pollination is not possible.

**Figure 9:**
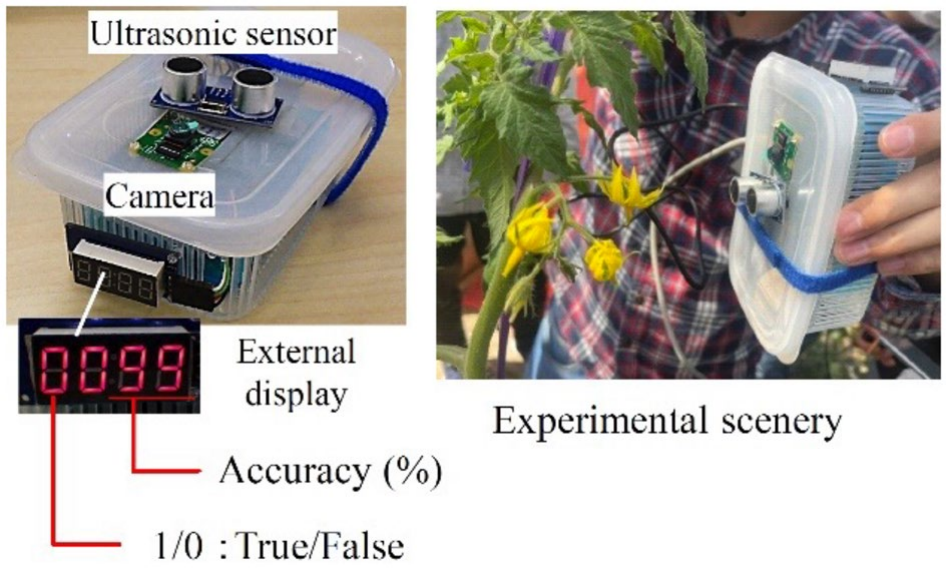
AI software implementation. An example and an image of an experimenter performing the experiment.

**Table 2:** Analysis accuracy of AI image classification machine.

	Pollination flowers	Non-pollination flowers
Flowers judged	108	86
Errors	1	5
Correct answer rate (%)	99.07	94.18

Correct answer rate = 1 - (errors/flowers judged).

**Figure 10:**
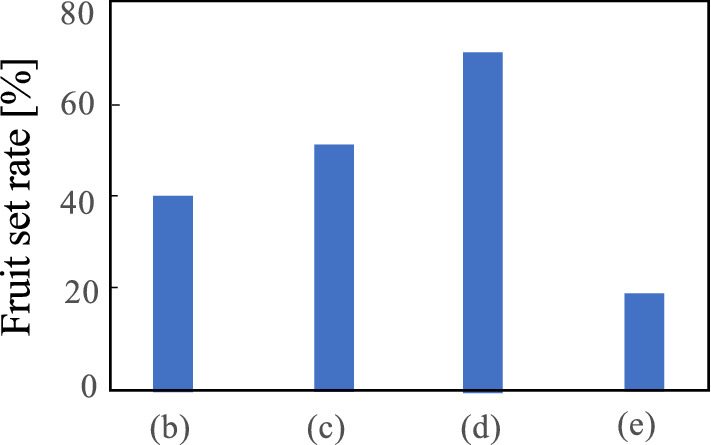
Fruit setting rate for each type of flower shape.

**Figure 11:**
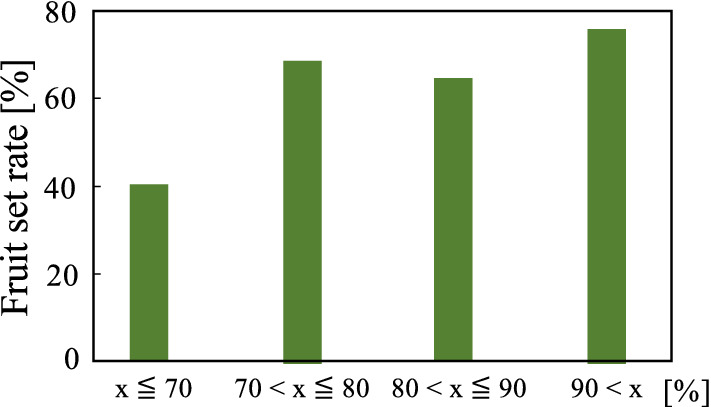
Threshold of the accuracy rate obtained from fruiting rate.

In this experiment, we aimed to confirm the performance of the AI image classification machine with simple information was displayed on the external display. When the pollination system in Fig. 1 was actually used, the output of the identification result notifies the robot or drone to undertake pollination. Conversely, when the output is 0, the operation resumes searching for ripe flowers. A photograph of the AI image classification machine in the experimental location is provided in Fig. 9. The displayed result of 1 or 0 allows us to assess the accuracy rate in real time using the LED external display. Therefore, the experimenter can visually confirm the data during the experiment. Moreover, when 1 is the output the accuracy rate is $\ge$ 70%. When the output is 0 the accuracy rate is $\le$ 70 %. This accuracy rate is intentionally relatively low to represent actual field operational conditions. We randomly selected 200 flower clusters were assessed using the AI image classification machine. If the output was 1 on the display, the pollination process was performed by physically vibrating the flower. The captured images, accuracy rate, and results (1/0) were saved separately in the internal memory and analyzed after the experiment. In addition, we marked the selected and pollinated flowers and continuously observed them to verify successful fruiting. The accuracy of the AI image classification machine is provided in Table 2. The fruiting rate is provided in Fig. 10. The figure provides the ratio of the number of setting fruits to the number of pollinated flowers. Fig. 11 provides the accuracy rate of the AI image classification machine with the fruiting rate.

## Discussion

**Figure 12:**
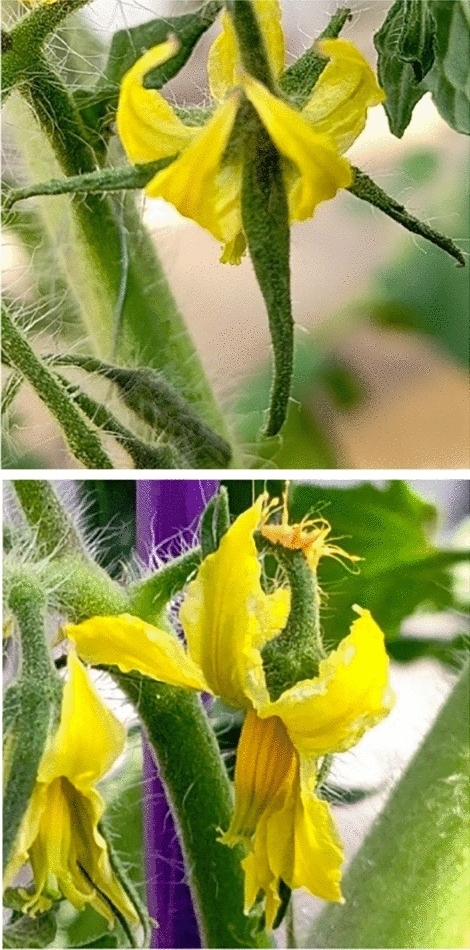
Examples of ambiguous flower shapes.

The accuracy of the AI image classification machine is provided in Table 2. The number of misclassified flowers was 6 flowers in approximately 200 flowers, resulting in a misclassification rate of approximately $\le$ 5%. This misjudgment was due to the camera shaking during photography causing the flower to be misjudged by the ambiguous and indeterminate shapes, or altered with sunlight effects in the greenhouse. Therefore, the image analysis performed well. However, when performing the image analysis, attention must be paid to deal with light. An example of flowers with uncertain shapes degrading accuracy is provided in Fig. 12. Even when visually confirmed, these ambiguous shaped flowers don’t consistently pollinate. The fruiting rate results provided in Fig. 10 are compared with the desktop predictions in Table 1. We confirmed the flower shape (d) provides the highest accuracy rate. The fruit setting rate was $\ge$ 70 %, confirming the flower shape in (d) is the most suitable for pollination.

The results confirmed successful pollination using the AI image classification machine. Therefore, we expect the AI image classification machine will sufficiently perform even when installed in a robot in an agricultural field. Figure 11 provides the success rate against the accuracy rate. When the accuracy rate of the AI image classification machine is below 70 %, the success rate is approximately 40 %. Conversely, when the accuracy rate of the AI image classification machine was 70 % or above, the fruiting rate was 60% or higher. Therefore, if the output accuracy rate is 70 % or above, the robot will correctly identify flowers ripe for pollination. The threshold of the AI image classification machine was set at $\ge$ 70%, which should provide sufficient pollination in real agricultural situations.

**Figure 13:**
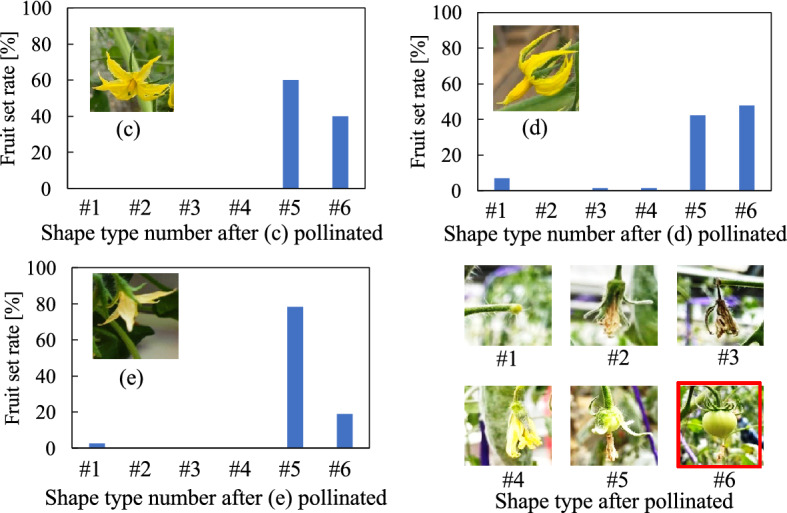
Fruit quality of the pollination experiment.

The relationship between the shape of the pollinated flower and the shape of the fruit set is demonstrated in Fig. 13. The flower shapes were classified from types #1 to #6 using the fruiting results. When the artificially pollinated flower shapes were (c), (d) or (e), they produced relatively high fruiting rates. The highest quality fruit shape was set by #6 shaped flowers. Thus, the fruit setting rate of #6, (c) was 40 %, (d) was 50 %, and (e) was 19 %. From these results, we identified flower shape (d) provides the highest fruit setting rate and also forms the highest quality fruit.

Image classification accuracy of AI by machine learning is generally required to be close to 100% classification accuracy, when applied to medical and engineering fields. However, we considered sufficient if the accuracy rate of 70% or more is obtained for the shape of flowers that can be pollinated, as shown in the result of Fig. 11. The reason is that not only the flower shape of Fig. 2 (d) can be pollinated. Of course, the flower shape (d) has the highest fruiting success ratio, but the (c) and (e) shapes can also fruit. The fuzzy accuracy rate is effective for the performance of the AI classifier system to improve the tomato yield.

In point of classification accuracy, in order for this technology to be installed in various robots in the future, it is desirable to extend machine learning by the transfer learning (TL) and Fine Tuning technology^23,24^. TL or Fine Tuning can be transferred or reused based on the original deep learning. Conventionally, when the growing season or the type of tomato is different, the shape of the flower changes slightly, so new machine learning is required. However, in the future, we hope to advance to a technology with a wider range of versatility by adding technologies such as TL and Fine Tuning. TL and Fine Tuning technology are positioned as extensions of this research, and we will be a future works.

### Possibility of implementing to small drones with low-resolution cameras

**Figure 14:**
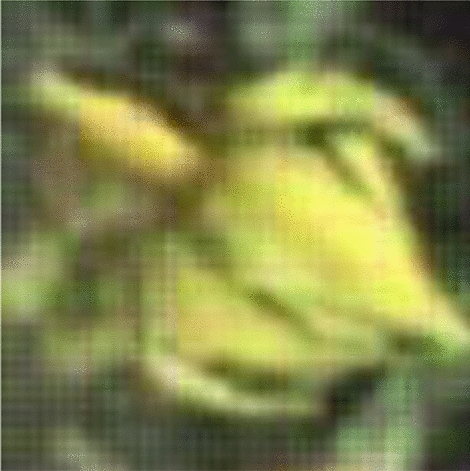
Low-resolution camera images intended for implementation on ultra-compact drones.

In order to consider the performance of the AI classification machine developed in this paper, we performed verification with a small camera for reference. Machine learning using CNN was performed assuming camera images mounted on ultra-small drones. The resolution of the camera used in the experiment in Fig. 9 is 1920 pixels × 1080 pixels, while the camera mounted on the ultra-small drone is 276 pixels × 196 pixels, which is about 1/6 the resolution. Figure 14 is the flower shape of (d) at this resolution. In the result of using AI image classification machine, the classification result of 88.7% was obtained. The classification result of the camera image used in the experiment was 95% or more, so this result was about 7% lower. However, since the accuracy rate of 70% or more is obtained, sufficient accuracy will be obtained even if the low-resolution camera for ultra-small drones is used. The reason for this result is machine learning was performed on images with Gaussian filter applied, and the high accuracy rate was obtained even when the image was blurred.

## Conclusion

We developed an AI image classification machine using CNN machine learning for a robot to determine the shape of tomatoes ready for pollination without the need for humans or bees. The image analysis algorithm was implemented as an AI image classification machine and its effective operation was confirmed in a tomato growing greenhouse. Pollination was only performed on ripe flowers. We compared the fruiting rate of the artificially pollinated tomatoes. When the accuracy rate of the AI image classification machine was set at 70% or above, the practical use of the pollination robots is realized. Moreover, we also identified the flower shape that yielded the highest fruiting rate and the highest quality fruit shape.

## Data Availability

The datasets used and/or analyzed during the current study are available from the corresponding author on reasonable request.
